# Transcriptome analysis reveals mechanism of early ripening in Kyoho grape with hydrogen peroxide treatment

**DOI:** 10.1186/s12864-020-07180-y

**Published:** 2020-11-11

**Authors:** Da-Long Guo, Zhen-Guang Wang, Mao-Song Pei, Li-Li Guo, Yi-He Yu

**Affiliations:** 1 College of Forestry, Henan University of Science and Technology, Luoyang, 471023 Henan Province China; 2 Henan Engineering Technology Research Center of Quality Regulation and Controlling of Horticultural Plants, Luoyang, 471023 Henan Province China

**Keywords:** Early ripening, Grape, Kyoho, Hydrogen peroxide, RNA sequencing

## Abstract

**Background:**

In a previous study, the early ripening of Kyoho grape following H_2_O_2_ treatment was explored at the physiological level, but the mechanism by which H_2_O_2_ promotes ripening at the molecular level is unclear. To reveal the molecular mechanism, RNA-sequencing analysis was conducted on the different developmental stages of Kyoho berry treated with H_2_O_2_.

**Results:**

In the comparison of treatment and control groups, 406 genes were up-regulated and 683 were down-regulated. Time course sequencing (TCseq) analysis showed that the expression patterns of most of the genes were similar between the treatment and control, except for some genes related to chlorophyll binding and photosynthesis. Differential expression analysis and the weighted gene co-expression network were used to screen significantly differentially expressed genes and hub genes associated with oxidative stress (*heat shock protein*, *HSP*), cell wall deacetylation (*GDSL esterase/lipase*, *GDSL*), cell wall degradation (*xyloglucan endotransglucosylase/ hydrolase, XTH*), and photosynthesis (*chlorophyll a-b binding protein, CAB1*). Gene expression was verified with RT-qPCR, and the results were largely consistent with those of RNA sequencing.

**Conclusions:**

The RNA-sequencing analysis indicated that H_2_O_2_ treatment promoted the early ripening of Kyoho berry by affecting the expression levels of *HSP, GDSL, XTH,* and *CAB1* and- photosynthesis- pathways.

**Supplementary Information:**

The online version contains supplementary material available at 10.1186/s12864-020-07180-y.

## Background

Grape (*Vitis vinifera* L.) is one of the oldest and most important fruit crops and is cultivated worldwide [1] Globally, grape berries, raisins, and wine are economically important commodities [2]. The grape berry ripening process is complicated and highly coordinated [3], with many physiological changes [4], including cell division and elongation, change in skin color, berry softening, increasing sugar content, decreasing organic acid and tannin contents, and the accumulation of aromas [5]. In addition, fruit ripening is an oxidative process, in which the redox homeostasis changes in cells [6]. Reactive oxygen species (ROS) play a key role in fruit ripening by regulating antioxidant systems [7, 8]. Reactive oxygen species initiate senescence for fruit ripening through the accumulation of O_2_ $\overline{\cdotp}$ and H_2_O_2_ [9, 10].

Hydrogen peroxide (H_2_O_2_) is an oxidant that can increase oxidative damage, cause metabolic dysfunction, and damage cell integrity [11]. At low concentrations, H_2_O_2_ can be used as signaling molecules that play key roles in biotic and abiotic stress responses, hormonal responses, growth and development, and fruit ripening [12]. However, when accumulated in plant tissues or fruits, H_2_O_2_ can promote programmed cell death [13]. Hydrogen peroxide not only functions in programmed cell death [14] but also is involved in the ripening of tomato [9] and pear [15]. Pilati et al. [16] observed a distinct oxidative burst during grape ripening and the rapid accumulation of H_2_O_2_, which was associated with grape softening and ripening. In addition, H_2_O_2_ is involved in the degradation of cell membranes and cell walls in tomato, thereby causing tomato softening and ripening [17]. Compared with a mutant (*rin*) tomato, the activity of ascorbate peroxidase decreased in wild-type tomato, and because H_2_O_2_ could not be cleared, it accumulated, thereby intensifying the degradation of cell walls and accelerating the softening and ripening of tomato [18].

The grape cultivar Fengzao is a bud mutant of Kyoho grape that has the distinct character of early ripening [19], maturing 30 d earlier than Kyoho. The comparison of Fengzao and Kyoho berries at the transcriptional level showed that ROS metabolism-related genes were significantly differentially expressed [20]. Differences were also revealed between the two cultivars in ROS metabolism at the physiological level [21]. Furthermore, H_2_O_2_, as an exogenous ROS, was sprayed on young Kyoho berries, which promoted earlier ripening, 20 d earlier than the control [10]. The treatment of hydrogen peroxide is not spraying on the whole plant, we just sprayed on the berry cluster. And there is not any obvious effect the growth and development of whole plant after the treatment. Although the exogenous H_2_O_2_ promoted berry ripening, the regulatory mechanism of this process at the molecular level is unclear.

In this study, to determine the influences of H_2_O_2_ treatment in promoting the early ripening of Kyoho grape, transcriptomic analysis was used to investigate and identify differentially regulated genes after H_2_O_2_ treatment. The results will provide the basis for the further exploration of the effects of H_2_O_2_ treatment on early ripening of grape berries.

## Results

### Overview of RNA-sequencing analysis

The transcriptome using total mRNA sequencing data was generated on an Illumina HiSeq 2500 platform (Illumina, San Diego, CA, USA). Approximately 46 million raw reads were obtained for each stage after the filtering of the raw reads. The Q_30_ values averaged approximately 94%. The clean reads were mapped to the grape reference genome (ftp://ftp.ensemblgenomes.org/pub/plants/release-38/fasta/vitis_vinifera). The mapping rate of the 23 samples to the grape reference genome was approximately 90% (Supplemental Table S1).

**Fig. 1:**
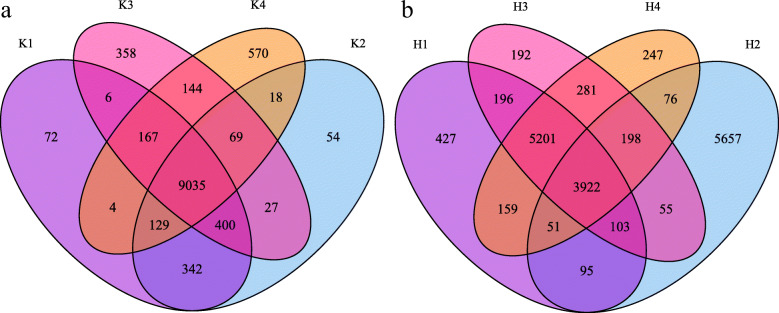
The number of expressed genes at difference sampling stages and the demonstration of the principal component analysis. **a** Venn diagram showed the number of the expressed genes in the control group; **b** Venn diagram showed the number of the expressed genes in the treatment group

To survey the distribution of gene numbers, a Venn diagram was prepared in the VennDiagram package (Fig. 1). A total of 40,568 genes were identified in the control groups, of which 10,154 were in stage K1, 10,073 in stage K2, 10,206 in stage K3, and 10,135 in stage K4, and 9035 genes were commonly expressed in all the control groups (Fig. 1a). A total of 40,591 genes were expressed in the treatment groups, of which 10,153 were in stage H1, 10,157 in stage H2, 10,147 in stage H3, and 10,134 in stage H4, and 3922 genes were commonly expressed in all the treatment groups (Fig. 1b). The K21 sample was eliminated from the following analysis due to the poor repetition.

### Overall expression patterns between the control and the treatment

**Fig. 2:**
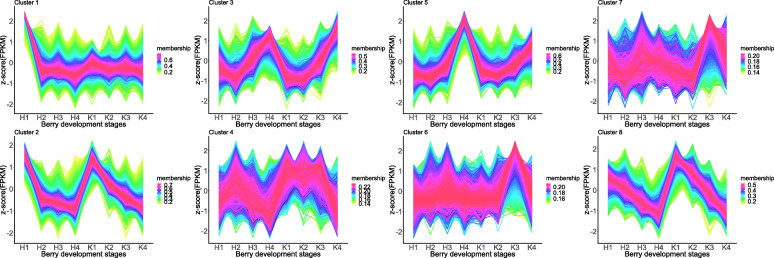
Cluster analysis of the gene expression patterns in the berry of the control and the treatment of ‘Kyoho’ across berry developmental stages. Clustering was performed based on TCseq analysis and the number of genes included in each of the clusters is indicated on the top of the figure. The Y-axis represents the FPKM values using 2 as the log base of a gene at different developmental stages. The X-axis represents the berry development. H and K represent the treatment with 300 mmol/L H_2_O_2_ and the control, respectively

To investigate the gene expression profiles of both the control and treatment, the expression patterns were compared using the TCseq clustering method. The division of clusters was determined using the vegan package. The highest Calinski criterion value was eight, indicating that the optimal number of clusters was eight. Hence, the gene expression patterns of the control and treatment groups were divided into eight categories (Supplemental Fig. S1). In the comparison of the gene expression profiles between the control and the treatment, the gene expression profiles of most clusters were similar (Fig. 2). However, the expression patterns between the control and the treatment in cluster 1 (4741 genes) and cluster 5 (4009 genes) were different. In cluster 1, the expression of genes decreased rapidly from the H1 to H2 stage. However, the expression patterns of genes were steady from the K1 to K4 stages. In cluster 5, the gene expression was similar in H1 and H2 stages and in K1 and K2 stages. The expression of genes increased rapidly from the H3 to H4 stage, but the increase was not as great from the K3 to K4 stage.

**Fig. 3:**
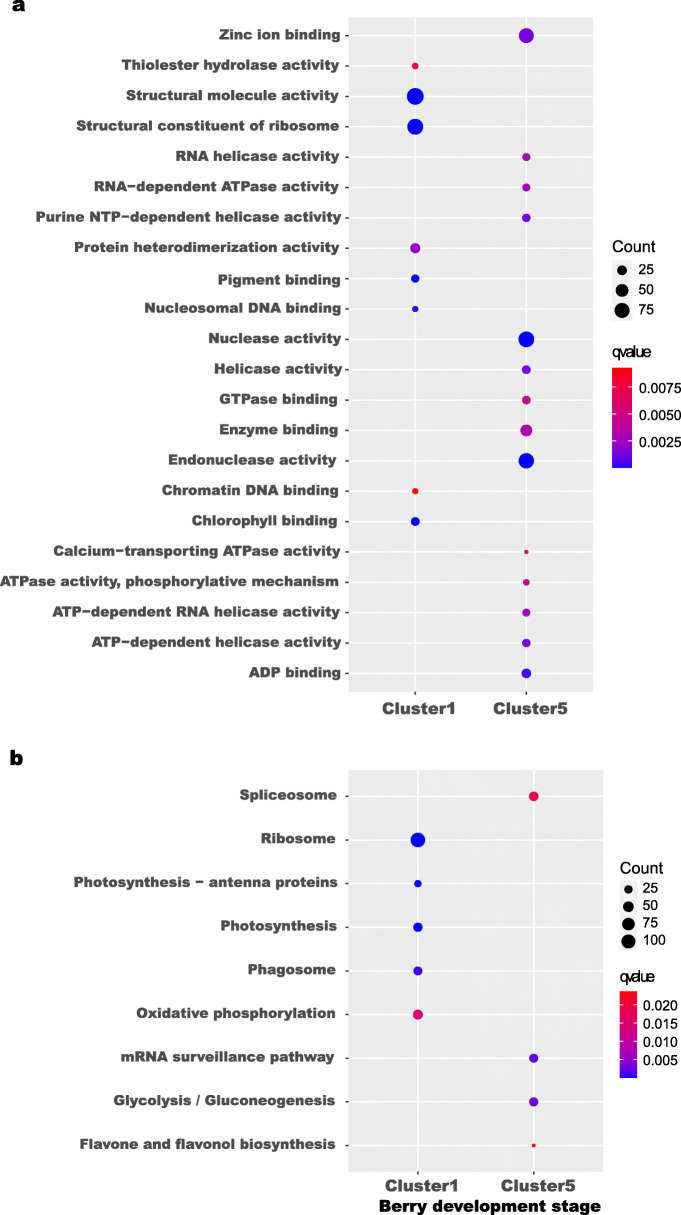
Scattergram of both GO enrichment analysis and KEGG pathways analysis of cluster 1 and cluster 5 which were different between the control and the treatment from Fig. 2 based on TCseq. The X-axis indicates the berry development stages; the Y-axis indicates the GO terms or KEGG pathway. **a** GO enrichment analysis of TCseq; **b** KEGG enrichment analysis of TCseq; Coloring indicates q-value with higher in red and lower in blue. And the lower q-value indicates the more significantly enriched. Point size indicates genes number

To further identify the functions of the genes in clusters 1 and 5, GO (Gene Ontology) and KEGG (Kyoto Encyclopedia of Genes and Genomes) enrichment analyses were performed (Fig. 3a and b) with ClusterProfiler. The GO enrichment analysis generated 22 top GO terms (Fig. 3a). Eight of the 22 top GO terms (e.g., structural constituent of ribosome, structural molecule activity, pigment binding, chlorophyll binding) were associated with cluster 1. The other 14 most enriched GO terms (e.g., nuclease activity, enzyme binding, endonuclease activity) were associated with cluster 5. The KEGG pathway analysis revealed the enrichment of nine pathways (Fig. 3b). Five of the nine top KEGG pathways (ribosome, photosynthesis, photosynthesis-antenna proteins, phagosome, and oxidative phosphorylation) were associated with cluster 1. The other four pathways (spliceosome, mRNA surveillance pathway, glycolysis/gluconeogenesis, and flavone and flavonol biosynthesis) were associated with cluster 5. Notably, the GO terms pigment binding and chlorophyll binding and the KEGG pathways photosynthesis-antenna proteins, photosynthesis, and oxidative phosphorylation are consistent with previous results [22]. Therefore, the genes associated with these GO terms and KEGG pathways were the focus of further analysis.

### Analysis of differentially expressed genes

**Fig. 4:**
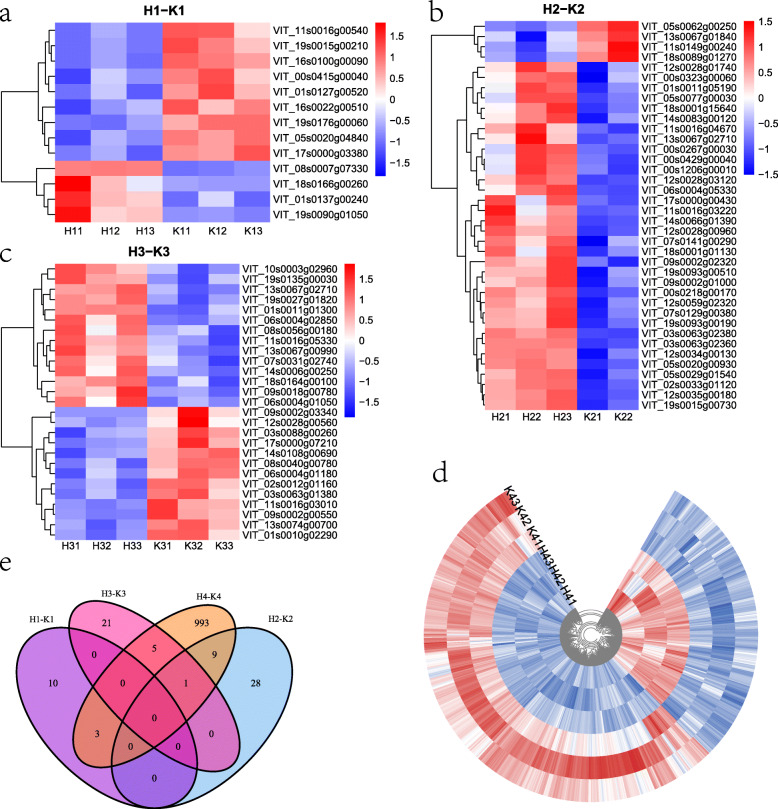
Heatmap showing the expression levels of DEGs both the treatment and the control, Venn diagram showing the number of the DEGs. **a** The DEGs expression from the H1-K1. **b** The DEGs expression from the comparison of H2-K2. **c** The DEGs expression from the comparison of H3-K3. **d** The DEGs expression from the comparison of H4-K4. **e** The number of DEGs from the each pair of comparison. Each column represents an experimental condition and each row represents a gene and three such replicates. Red means up-regulated and blue means down-regulated

In this study, the differentially expressed genes (DEGs) were identified using DESeq2. A total of 1089 DEGs were identified in the comparisons of the treatment and control groups. Some DEGs were selected on the basis of the rank of the fold changes of up- or down-regulation and are listed in Table 1. To further explore the expression of DEGs in the control and treatment groups, a heat map was constructed (Fig. 4) with the pheatmap package. Figure 4 reveals that the DEGs were distributed in the comparisons of H1 and K1 (four up-regulated and nine down-regulated), H2 and K2 (34 up-regulated and four down-regulated), H3 and K3 (14 up-regulated and 13 down-regulated), and H4 and K4 (354 up-regulated and 657 down-regulated) stages (the gene IDs are shown Supplemental Table S2). To identify the DEGs in common, a Venn diagram was constructed; however, the treatment and the control did not share any of the same genes (Fig. 4e).

**Table 1:** List of differentially expressed genes for the treatment over the control after H_2_O_2_ treatment

Stage	Gene ID	Log2FoldChange	padj	Up/Down	Uniprot:Description
H1-K1	VIT_19s0090g01050	4.580139553	0.001810767	up	Endoglucanase 11
	VIT_01s0137g00240	3.347436354	0.049115888	up	Probable pectate lyase 5
	VIT_01s0127g00520	−1.333128782	0.019028882	down	Probable nucleoredoxin 1
	VIT_05s0020g04840	−1.42318995	0.019028882	down	GDSL esterase/lipase 7
	VIT_00s0415g00040	−1.691361575	0.03689468	down	Glycine-rich domain-containing protein 1
	VIT_16s0022g00510	−1.891347211	0.022140035	down	23.6 kDa heat shock protein, mitochondrial
	VIT_17s0000g03380	−2.95452543	0.02214004	down	Protein SAR DEFICIENT 1
	VIT_19s0015g00210	−3.642741092	1.13E-06	down	Zinc transporter 2
	VIT_11s0016g00540	−3.777944529	6.65E-06	down	Respiratory burst oxidase homolog protein E
	VIT_16s0100g00090	−7.717325156	0.00881588	down	Peroxidase 66
H2-K2	VIT_05s0029g01540	2.301497625	2.57E-08	up	Protein ACCELERATED CELL DEATH 6
	VIT_11s0016g03220	2.242129946	0.0062515	up	Probable RNA-dependent RNA polymerase 5
	VIT_12s0028g03120	2.120136837	0.0069495	up	Protein E6
	VIT_12s0034g00130	2.05843658	0.0001454	up	UDP-glucose flavonoid 3-O-glucosyltransferase 6
	VIT_17s0000g00430	1.720472715	0.00265815	up	Transcription factor bHLH137
	VIT_03s0063g02380	1.634636111	2.69E-11	up	Organ-specific protein S2
	VIT_05s0077g00030	1.610540187	0.005766187	up	Ammonium transporter 1 member 1
	VIT_18s0001g15640	1.420949969	0.017401315	up	Pathogen-related protein
	VIT_19s0015g00730	1.17834606	5.58E-05	up	Cellulose synthase-like protein E6
	VIT_02s0033g01120	1.08276489	6.76E-14	up	Probable methyltransferase PMT20
	VIT_05s0062g00250	−2.248382917	2.69E-11	down	Xyloglucan endotransglucosylase/hydrolase 2
	VIT_18s0089g01270	−1.677685736	1.84E-07	down	22.0 kDa class IV heat shock protein
H3-K3	VIT_19s0135g00030	2.807368996	0.027112354	up	Caffeic acid 3-O-methyltransferase
	VIT_18s0164g00100	2.740316141	0.027112354	up	Laccase-14
	VIT_14s0006g00250	1.461809302	0.023518312	up	Cysteine-rich repeat secretory protein 60
	VIT_11s0016g05330	1.345232867	0.002817032	up	SPX domain-containing protein 1
	VIT_13s0067g00990	1.032137018	0.01657462	up	Protein SMAX1-LIKE 7
	VIT_11s0016g03010	−1.21314274	0.003481905	down	Probable glutamate carboxypeptidase 2
	VIT_09s0002g00550	−1.544214265	0.000897028	down	GDSL esterase/lipase 1
	VIT_01s0010g02290	−1.614256666	0.00078407	down	25.3 kDa heat shock protein, chloroplastic
	VIT_06s0004g01180	−1.753589771	0.018816772	down	Peroxidase 15
	VIT_02s0012g01160	−2.026019129	0.018816772	down	Protein NRT1/ PTR FAMILY 7.3
H4-K4	VIT_04s0079g00690	8.803363773	2.24E-25	up	Glutathione S-transferase F12
	VIT_18s0164g00100	3.248615391	1.11E-05	up	Laccase-14
	VIT_01s0011g06290	1.43663321	4.54E-13	up	Purple acid phosphatase 3
	VIT_19s0015g00210	1.332996886	0.028360846	up	Zinc transporter 2
	VIT_09s0002g00550	−1.796281864	8.20E-07	down	GDSL esterase/lipase 1
	VIT_18s0089g01270	−1.829201669	0.022374842	down	22.0 kDa class IV heat shock protein
	VIT_16s0022g00510	−2.41607704	2.34E-05	down	23.6 kDa heat shock protein, mitochondrial
	VIT_10s0003g02890	−3.111297081	5.87E-62	down	Chlorophyll a-b binding protein 40, chloroplastic
	VIT_02s0154g00480	−3.601988523	4.54E-13	down	Small heat shock protein, chloroplastic
	VIT_08s0058g00210	−5.111929687	0.016649888	down	17.3 kDa class I heat shock protein
	VIT_17s0000g03940	−5.756448165	0.000924922	down	Cytochrome P450 CYP736A12
	VIT_07s0031g01710	−6.950745362	0.000216632	down	Probable WRKY transcription factor 51

**Fig. 5:**
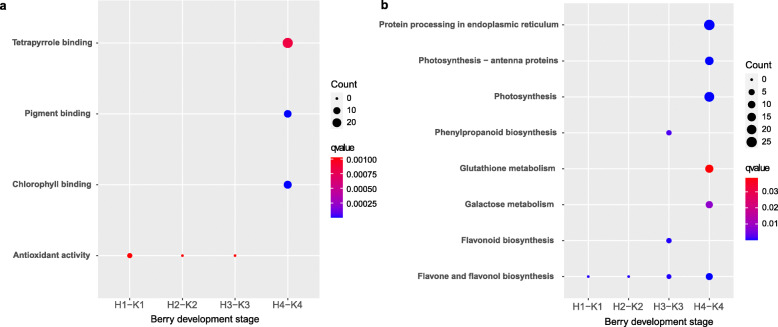
Scattergram of both GO enrichment analysis and KEGG pathways analysis. The X-axis indicates the berry development stages; the Y-axis indicates the GO terms or KEGG pathway. **a** GO enrichment analysis of DEGs from each pair of comparison. **b** KEGG pathway analysis of DEGs from each pair of comparison. Coloring indicates q-value with higher in red and lower in blue. And the lower q-value indicates the more significantly enriched. Point size indicates genes number

To further investigate the molecular mechanisms involving the DEGs in the treatment and the control, the DEGs were analyzed by GO and KEGG enrichment analyses (Fig. 5). The GO enrichment analysis revealed four top GO terms, including pigment tetrapyrrole binding, pigment binding, chlorophyll binding, and antioxidant activity (Fig. 5a). The GO enrichment analysis produced no results for the H2-K2 and the H3-K3 comparisons. The KEGG pathway analysis identified eight top pathways, which included, for example, photosynthesis-antenna proteins, protein processing in endoplasmic reticulum, and photosynthesis (Fig. 5b). The KEGG enrichment analysis produced no results for the H1-K1 and the H2-K2 comparisons. Notably, pigment binding, chlorophyll binding, and photosynthesis were significantly enriched in the H4-K4 comparison, which are results consistent with those in Fig. 3. The results suggested that the H_2_O_2_ treatment affected the expression of genes associated with photosynthesis and pigment binding.

### Weighted gene co-expression network analysis

**Fig. 6:**
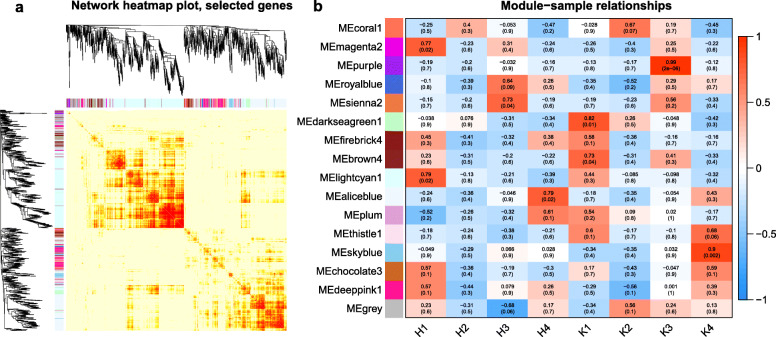
Gene modules were identified by a color name (MEcolornumber) as assigned by the weighted gene co-expression network analysis (WGCNA) package. **a** Heatmap plot of topological overlap in the gene network. **b** Heatmap correlation of berries’ differential genes of each of 16 gene modules. In the heat map, each row and column corresponds to a gene, a light color denotes low topological overlap, and progressively darker red denotes a higher topological overlap. Darker squares along the diagonal correspond to modules. The gene dendrogram and module assignment are shown along the left and top

In this study, weighted gene co-expression network analysis (WGCNA) was conducted on the transcriptomic data sets from Kyoho berries in the H_2_O_2_ treatment. The analysis was conducted using the WGCNA package in R. The modules of highly correlated genes were identified based on the fragments per kilobase per million mapped reads (FPKM) value. Figure 6a shows the heat map of the gene co-expression network. In the figure, the left and top measurements are the results of the gene system cluster tree and the gene network/module analyses, respectively. However, in the heat map at the bottom right, the regions represent the dissimilarity between genes, and the smaller the value is, the darker the color. Generally, with the same genes between modules, the color was darker; whereas the color between modules was lighter. The co-expression modules were constructed via Pearson’s correlation coefficient for gene expression across all samples, and 16 modules were constructed (Fig. 6b). Each cell in Fig. 6b is colored based on the statistical significance and is labeled with two numbers, with the upper number the correlation coefficient and the lower number the *P-*value. Based on the criteria of *P* ≤ 0.05 and a Pearson’s correlation coefficient greater than 0.7, as shown in Fig. 6b, the modules MEmagenta2, MEsienna2, MEaliceblue, MEdarkseagreen1, MEpurple, and MEskyblue were selected at the H1, H3, H4, K1, K3, and K4 stages, respectively.

To further understand the functions of genes in the modules, GO and KEGG enrichment analyses of each module were conducted to identify its involvement in particular biological processes and metabolic pathways (Supplemental Fig. S4). The top 25 GO terms from the GO enrichment analysis are shown in Supplemental Fig. S4a. Among the GO terms, 21 were in the H1 stage and included, for example, structural constituent of ribosome, structural molecule activity, and microtubule motor activity. Zinc ion binding and nuclease activity were enriched in stage H4; and structural constituent of nuclear pore and glutathione transferase activity were enriched in stages K1 and K3, respectively. The GO enrichment analysis produced no results in stages H3 and K4. The results indicated that the H_2_O_2_ treatment might primarily affect the expression of genes in early stages. A total of 14 KEGG pathways were enriched (Supplemental Fig. S4b). Six pathways, which included, for example, ribosome, protein export, proteasome, DNA replication, and biotin metabolism, were enriched in stage H1. The four pathways of mRNA surveillance pathway, glycolysis/gluconeogenesis, fatty acid degradation, and arachidonic acid metabolism were enriched in stage H4 stage. The other enriched pathways of glutathione metabolism, protein processing in endoplasmic reticulum, chlorophyll metabolism, and one carbon pool by folate were in stages K3 and K4. The KEGG enrichment analysis produced no results in stages H3 and K1. To summarize, the H_2_O_2_ treatment primarily affected the expression of genes in early berry developmental stages (Supplemental Fig. S4).

### Reconstruction of gene co-expression network

**Fig. 7:**
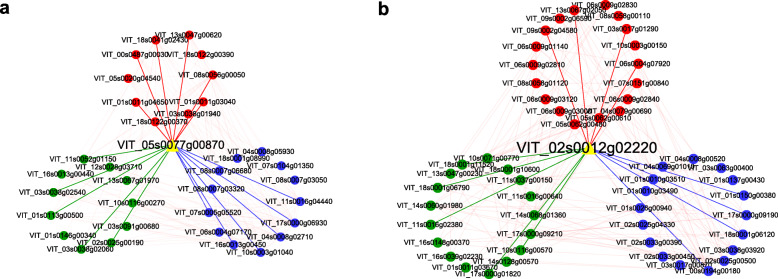
Cytoscape representation co-expressed genes with edge weight ≥ 0.10. The important hub gene was noted with yellow. **a** The hub gene of the H3 stage. **b** The hub gene of the H4 stage, respectively

Hub genes were identified by sorted K_ME_ values, which indicated the eigengene connectivity in the WGCNA analysis [23]. The co-expression network of individual co-expression modules was visualized using Cytoscape software [24]. In this study, some hub genes were selected from the highest K_ME_ values of each significantly different module. Based on the results of a previous study [22], the focus was on the hub genes *GDSL* (GDSL esterase/lipase, VIT_05s0077g00870) and *XTH30* (xyloglucan endotransglucosylase/hydrolase protein 30, VIT_02s0012g02220). Figure 7 shows that *GDSL* and *XTH30* included 17 and 11 edges, respectively, and all gene annotations are shown Supplemental Table S3. Supplemental Fig. S2 shows the networks of H1, K1, K3, and K4, and the annotation of the respective hub genes was as uncharacterized protein; hypothetical zinc ion binding protein, hypothetical protein; udp-glucose flavonoid 3-o-glucosyltransferase 6-like, probable glutathione s-transferase; 18.2 kDa class I heat shock protein; and 18.6 kDa class III heat shock protein.

### Verification of differentially expressed genes and hub genes by RT-qPCR

**Fig. 8:**
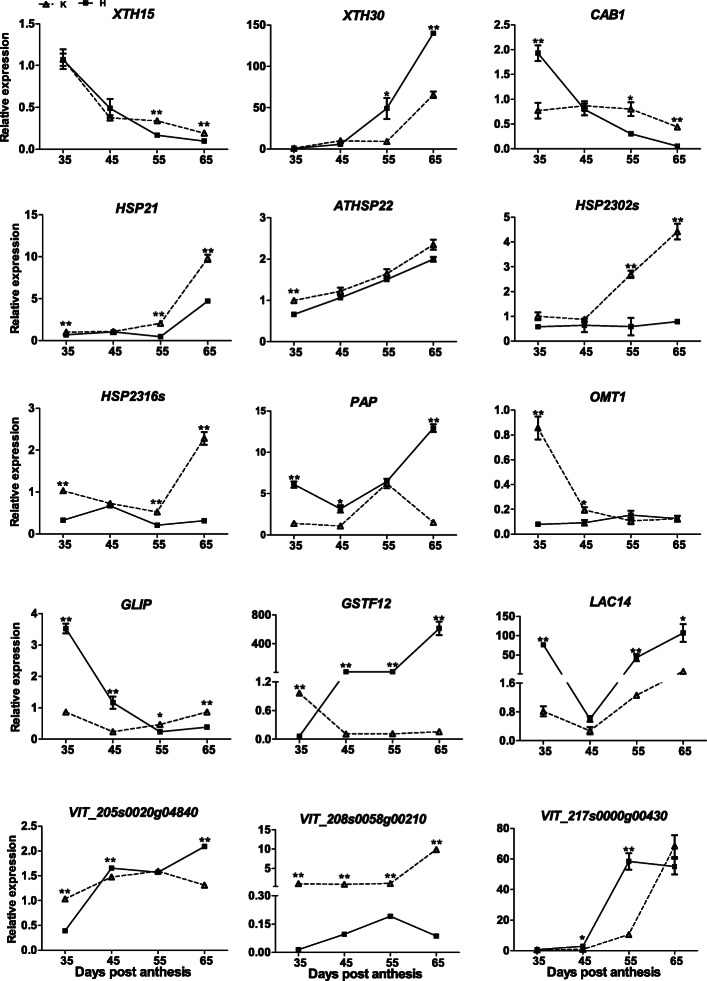
qRT-PCR expression patterns of both 14 DEGs and one hub genes detected in the RNA-Seq profiles of the control and the treatment. Three biological replicates are used for qRT-PCR validation. Significant differences between means were made using One-way ANOVA followed by Tukey’s HSD post hoc test. Error bars represented standard deviations from three independent biological replicates. The asterisk (_*_) stands for the levels of significant difference (*p value ≤0.05, **p value ≤0.01)

To verify the RNA-sequencing results of DEGs and hub genes, 14 DEGs (*XTH15, CAB1, HSP21, ATHSP22, HSP23, PAP, OMT1, GLIP, GSTF12, LAC14, VIT_205s0020g04840, VIT_208s0058g00210*, and *VIT_217s0000g00430*) and one hub gene (*XTH30*) were selected to perform RT-qPCR (Fig. 8; Supplemental Table. S5). In the comparison between the control and the treatment, the expression levels of *XTH15, CAB1, HSP21, HSP23*, and *VIT_208s0058g00210* in the treatment were significantly lower than those in the control at 55 days post anthesis (dpa). Compared with the control, the relative expression levels of *XTH30, GSTF12, LAC14*, and *VIT_217s0000g00430* were significantly higher in the treatment at 55 dpa. The relative expression profiles of *ATHSP22* were similar. The relative expression level of *GLIP* in the treatment was higher than that in the control at 35 and 45 dpa, whereas the relative expression level of *OMT1* in the treatment was lower than that in the control at 35 and 45 dpa. Thus, the results of RNA sequencing and RT-qPCR were largely consistent. Linear regression [(qRT-PCR value) = a (RNA-seq value) + b] analysis showed that the transcript abundance assayed by real-time PCR and the transcription profile revealed by RNA-seq data were highly correlated (R^2^ = 0.7874) (Supplemental Fig. S3).

## Discussion

Hydrogen peroxide is a crucial ROS in biological processes that contributes to tolerance against a variety of stresses [25, 26] and is also a secondary messenger that regulates the development and growth of plants to some degree [27]. In addition, according to previous results, Kyoho berries with H_2_O_2_ treatment ripened 20 days earlier than the control [10]. Nevertheless, the effect of H_2_O_2_ treatment on changes in the expression of genes of Kyoho is currently not understood. RNA sequencing can measure the expression level of each gene in a sample and also helps to reveal the expressional differences among different samples [28, 29]. Hence, RNA sequencing was used to identify the changes in gene expression after H_2_O_2_ treatment. We may miss these genes. That need the extensive sampling points. Furthermore, if this is true, the changes of assumed genes will affect a series of changes of other genes, its effect will not disappear, otherwise, it could not promote the early ripening of the grape, the present study primarily caught these. And we indeed collected the samples shortly after the treatment for the first sampling point.

A total of 1089 DEGs were identified in the RNA-sequencing data (Fig. 4). To determine the common DEGs in the comparison of the same stages, Venn diagrams were constructed (Fig. 4e). However, no DEGs were shared in the comparisons of the same stages. This result may be because few DEGs were identified in the early berry developmental stages. Based on the reasons given above, TCseq (Fig. 2) and WGCNA (Fig. 6) analyses were performed. The TCseq showed that most of the genes had similar expression profiles (Fig. 2), i.e., the H_2_O_2_ treatment did not cause dramatic changes in gene expression, although some key pathways might have been affected. GO enrichment analysis revealed that the pathway of chlorophyll binding was enriched, as shown in Fig. 3a and Fig. 5a; and the KEGG enrichment analysis revealed that photosynthesis-antenna proteins and photosynthesis pathways were enriched, as shown in Fig. 3b and Fig. 5b.

Chlorophyll is essential in photosynthesis [30], which has an important role in the physiological processes of plant growth and development [31]. Many studies demonstrate that oxidative stress can interfere with photosynthesis [32, 33], which causes an imbalance in the electron transport chain in chloroplasts, thereby accelerating the production of ROS [34] and causing oxidative damage [35], accelerating plant senescence [36]. In addition, Yu et al. [37] found that abiotic stress can destroy the photosynthetic apparatus, which results in changes in the expression levels of proteins associated with photosynthesis, reducing photosynthesis and causing early senescence. In this study, the pathway of chlorophyll binding was enriched in cluster 1 and in the comparison of stages H4-K4 (Fig. 3a and Fig. 5a), and the pathways of photosynthesis and photosynthesis-antenna proteins were enriched in cluster 1 (Fig. 3b) and in the comparison of stages H4 and K4 (Fig. 5b).

The role of photosynthesis in fruit development and ripening has been much discussed [38]. Therefore, the search for mechanisms that underlie the variability in photosynthesis during fruit ripening is an important direction in the research to better understand the process of fruit ripening [39]. At present, most of the works that examine photosynthesis in fruit during ripening involve tomato. According to Cocaliadis et al. [40], with the induction of higher photosynthesis during the green stages, tomato fruit ripening can be delayed. In addition, Piechulla et al. [41] found that photosynthesis decreases during tomato fruit ripening. Wang et al. [42] used combined metabolomic and transcriptomic analyses and also reported that photosynthesis is important in tomato fruit ripening. These studies show that although the peel of mature green fruits is photosynthetically active, there is a gradual decrease in the intensity of photosynthesis during ripening [43]. In this study, both GO enrichment and KEGG pathway analyses revealed the importance of the photosynthesis pathway (Fig. 3), and the genes associated with photosynthesis were screened for further analysis via DEG, Tcseq, and WGCNA analyses.

**Fig. 9:**
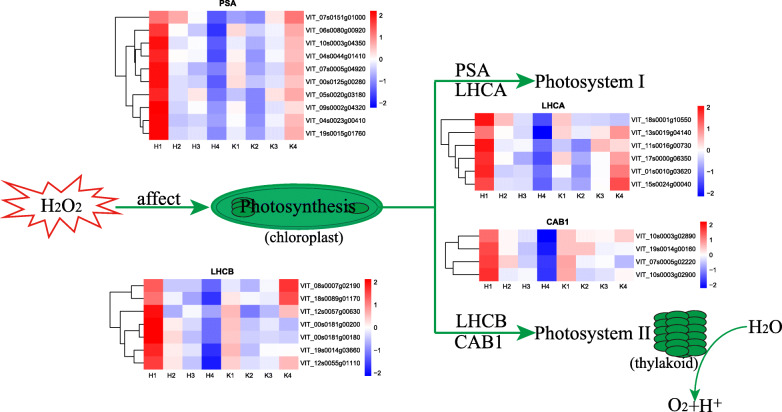
Heatmap showing the expression patterns of genes involved in the photosynthesis pathway both in the treatment and the control

The chlorophyll a/b-binding proteins (CABs) are the apoproteins of the light-harvesting complex of photosystem II (PSII) in higher plants [44]. CABs are normally part of the antenna complex [45]. According to Ma and Yang et al. [46], multiple genes encode CABs in higher plants. In a previous report, an increase in the expression of the *CAB* gene caused the accumulation of chlorophyll and stability in the chloroplast thylakoid membranes in green tomato fruit [47]. Silva et al. [48] found that the expression of *CABs* is affected by abiotic stress, which indicated the *CAB* gene plays an important role in abiotic resistance. Peralta et al. [49] reported that with an increase in the expression levels of *CAB1*, there is a lack of yellowing in rosette leaves, and therefore, the increase in the expression level delays leaf senescence. In addition, melatonin can delay senescence in kiwifruit leaves by maintaining the chlorophyll content [50]. When the expression of *CAB* is down-regulated, the kiwifruit leaves are in senescence. However, melatonin treatment increases the expression of the *CAB* gene and delays the senescence of kiwifruit leaves [50]. In this study, *CAB1* (VIT_10s0003g02890, chlorophyll a-b binding protein 40) was significantly down-regulated after H_2_O_2_ treatment (Table 1), and the RT-qPCR results showed that the expression of the *CAB1* gene was significantly lower in the treatment than in the control at 55 dpa (Fig. 8). Notably, H_2_O_2_ treatment causes veraison of Kyoho at 55 dpa [10]. The above results indicated that the H_2_O_2_ treatment inhibited the expression of *CAB1* and thereby promoted early berry ripening. These results are consistent with those of Liang et al. [50]. In addition, other genes (*PSA*, *LHCB*, and *LHCA*) associated with photosynthesis were affected by the exogenous H_2_O_2_ treatment (Fig. 9), and these genes might also play a crucial role in early berry ripening.

Heat shock proteins (HSPs) are molecular chaperones that reduce the damage due to multiple stresses [51]. Driedonks et al. [52] found that the HSPs can also protect cells against abiotic stresses by interacting with ROS-scavenging genes. On the basis of protein size, amino acid sequence homology, and function, HSPs are classified into five subfamilies, including small HSPs (sHSPs) [53]. Hilton et al. [54] reported that HSPs represent the first line of defense in a cell to prevent protein misfolding. In addition, sHSPs are important defense-induced factors and play key roles not only in the response to oxidative stress but also in the response to drought, salt, cold, and heat stress [55, 56]. The overexpression of sHSPs protects photosystem II from oxidative stress in tomato and tobacco [57, 58]. Ji et al. [59] showed that *HSP* genes promoted the ripening of grape berries by using a gene family analysis, a structured phylogenetic tree, a subcellular localization study, and further functional analysis. In the RNA sequencing of tomato fruit, four differentially expressed *HSP* genes were identified, which were considered to play a key role in fruit ripening [60]. The *NJJS4* gene is a type of HSP20-coding gene, which participates in regulating strawberry fruit ripening [61]. In addition, Arce et al. [62] report that the sHSP gene family in tomato is related to fruit ripening, as indicated by the sequence-conserved promoter architecture. Moreover, an sHSP influences pectin depolymerization in tomato fruit ripening [63]. In a proteome-wide study, eight *HSP* genes were associated with tomato fruit development and ripening [64], demonstrating that *HSP* genes indeed participate in tomato fruit ripening [65]. Stress is a well-known stimulus that triggers the induction of sHSPs [62]. In particular, sHSPs are also induced during tomato ripening [66], suggesting that the stress induced affected the *HSP* genes, thereby causing tomato fruit ripening. In this study, the exogenous H_2_O_2_ treatment was equivalent to exogenous stress in the Kyoho grape berry and therefore affected the expression of *HSP* genes, which resulted in the berry ripening. In a previous study, riboflavin treatment affected the expression of *HSP* genes [22]. On the basis of that study, in this study, five DEGs were selected that were associated with HSPs: *ATHSP22* (VIT_18s0089g01270), *VIT_208s0058g00210* (VIT_08s0058g00210), *HSP23* (VIT_16s0022g00510, VIT_02s0154g00480), and *HSP21* (VIT_01s0010g02290). RT-qPCR was used to verify the expression of those genes (Fig. 8).

The expression of most *HSP* genes in the treatment group was significantly lower than that in the control group at 55 dpa (Fig. 8). However, with H_2_O_2_ treatment, Kyoho berries ripen 20 days earlier than those in in the control [10]. The results suggested that H_2_O_2_ treatment down-regulated the expression of *HSP* genes, which may increase the level of oxidative stress. Oxidative stress can be best assessed by the extent of lipid peroxidation catalyzed by lipoxygenase [67]. Catalase is one of the primary enzymatic defenses against oxidative stress induced by senescence [68], and when superoxide dismutase (SOD) decreases during fruit senescence, the O_2_ $\overline{\cdotp}$ concentration increases, thereby causing oxidative stress [69]. Lipoxygenase, catalase, and SOD are associated with ROS metabolism [70]. Oxidative stress can cause the accumulation of ROS, and by causing cellular membrane damage; the excess ROS participate in the process of fruit ripening [71, 72]. According to Chin et al. [73], control of oxidative stress in mango fruits can extend the shelf life in the postharvest stage. Thus, these results demonstrate that oxidative stress regulates fruit ripening by regulating ROS metabolism. By contrast, the overexpression of *HSP* genes can alleviate damage caused by abiotic stresses in *Arabidopsis* and rice, thereby delaying senescence [74, 75]. Previous results also indicate that exogenous ROS treatment can promote the early ripening of berries by affecting the expression *HSP* genes [10, 22]. However, the relationship between HSPs genes and berries ripening is unclear at present. i.e. it indeed could not distinguish “cause” and “effect”.

The GDSL esterases belong to a family of lipid hydrolysis enzymes in higher plants [76]. These esterases/lipases have been identified in grape (96 members), western balsam poplar (126), sorghum (130), and moss (57) [77]. The GDSL esterases/lipases have a crucial role in the regulation of plant growth and development, morphogenesis, synthesis of secondary metabolites, and defense response [78]. Fatty acids are the major energy reserve substance stored in the mature seeds of many higher plants and can regulate plant growth and development [79]. In the enrichment analysis, the pathway of fatty acid degradation was identified at stage H4 (Supplemental Fig. S4b). Huang et al. [80] report that the GDSL esterases/lipases can degrade fatty acids in mature *Arabidopsis* seeds. Acetylation modifies the cell wall and is beneficial to the formation of secondary wall architecture [81]. The GDSL esterases/lipases remove acetyl groups from the xylan backbone [81], and as a result, the enzymes promote the softening of cell walls by deacetylation. The GDSL esterases/lipases also play an important role in plant defense responses [82]. *GLIP1* exhibits antimicrobial activity and has positive roles in defense in *Arabidopsis* [83]. Gao et al. [84] found that the down-regulation of *GLIP1* increases rice resistance to pathogens, but with the overexpression of *GLIP1*, disease resistance decreases significantly in rice. The results suggest that the *GLIP1* has an important role in disease resistance and in the response to abiotic stress in rice. In addition, Ni et al. [85] show that *GLIP1* may be associated with grape ripening. Notably, in a previous study, ROS and pathogenesis-related (PR) genes had key roles during grape berry ripening, in addition to the genes of some cell wall-degrading enzymes [20].

The *GLIP1* (VIT_09s0002g00550) and *VIT_205s0020g04840* were the DEGs identified in the present study. The RT-qPCR revealed that the expression level of *GLIP1* in the treatment was significantly higher than that in the control at 35 and 45 dpa. However, at 65 dpa, the expression level of *GLIP1* in the treatment was significantly lower than that in the control (Fig. 8). It is just the assumption based on the published reports about the function of GLIP. GLIP1 was selected due to its differentially expression both in RNAseq and qRT-PCR analysis. We just showed the expression characterization which may be related. Of course, more proof is needed in the future.

Fruit softening is associated with the modification of xyloglucan [86], and xyloglucan plays a crucial role in loosening or stiffening the cell wall by binding to cellulose [87, 88]. Hemicellulose is a polysaccharide component of the primary cell wall, and the depolymerization of hemicellulose is responsible for fruit ripening [89]. Xyloglucan endotransglycosylase/hydrolases (XTHs) are cell wall enzymes with hydrolase and endotransglycosylase activities [90]. The XTHs can catalyze endolytic cleavage of xyloglucan polymers, thereby promoting fruit softening [86]. The *XTH* genes also participate in the degradation of cell walls [91] and are involved in fruit softening [92, 93]. Yun et al. [94] confirm that an *XTH* gene can promote the ripening of banana fruit by the degradation of hemicelluloses. Similarly, an *XTH* gene can cause the ripening and softening of kiwifruit [95].

In this study, *XTH15* (VIT_05s0062g00250) (Fig. 4b) and *XTH30* (VIT_02s0012g02220) (Fig. 7b) genes were selected. RNA sequencing showed that *XTH15* was significantly down-regulated in the treatment group (Table 1). Compared with the control, the expression level of *XTH15* was significantly lower in the treatment at 55 dpa. However, the expression level of *XTH30* was significantly higher in the treatment than that in the control at 55 dpa (Fig. 8).

**Fig. 10:**
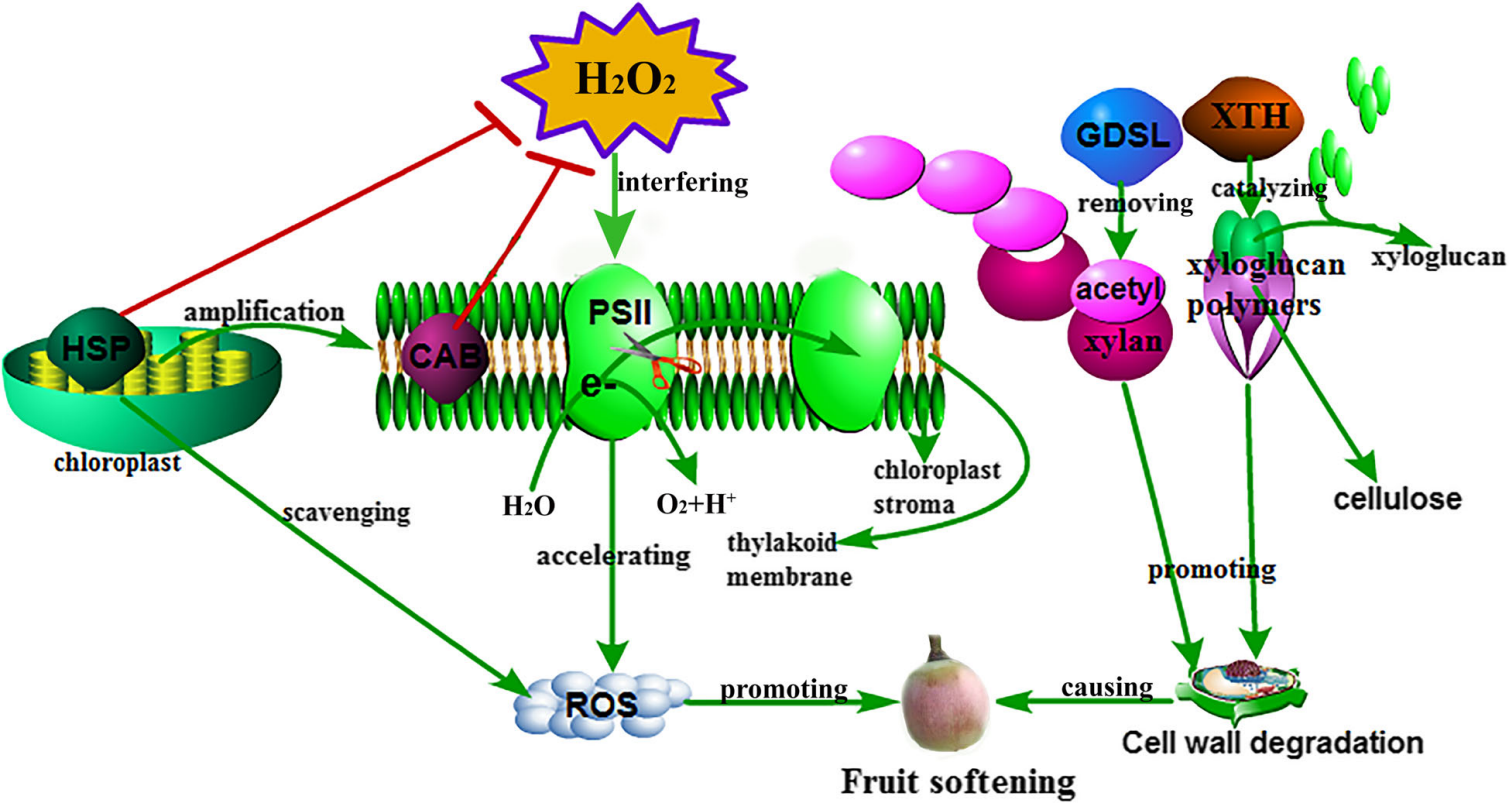
The presumed diagram of the DEGS (*XTH*, *HSP*, *GDSL* and *CAB1*) involved in fruit ripening

Shi et al. [96] found that XTH15 is strongly expressed in young organs of *Arabidopsis*, suggesting that it play roles in cell expansion, although hydrolytic activity was undetectable. This result suggests that XTH15 has only endotransglycosylase activity. Divol et al. [97] show that *XTH15* and *XTH30* are in different main groups, according to a gene family analysis. Thus, XTH15 and XTH30 may play different roles in fruit development and ripening. Yan et al. [98] show that the knockout of *XTH30* results in a lower level of H_2_O_2_ in the mutant than in the wild type, thereby alleviating oxidative damage in *Arabidopsis*. The result suggests that *XTH30* is associated with ROS. In this study, the expression level of *XTH30* was significantly higher in the treatment than in the control at 55 dpa. Therefore, the H_2_O_2_ treatment promoted the expression of *XTH30*, which increased the oxidative damage and caused early ripening of berries. The *XTH15* gene may only regulate cell expansion in early berry developmental stages, and therefore, the function of *XTH15* needs to be studied further. In addition, the relationship between XTH15 genes and berries ripening is unclear. The present study just demonstrated *XTH15* after the H_2_O_2_ treatment. The clarification of cause or effect need more further study to explore it. We will do some transgenic study to test it in the future. To summarize, the H_2_O_2_ treatment interfered with photosystem II, causing the accumulation of ROS and thereby promoting the ripening of fruit. The genes *GDSL* and *XTH* promoted cell wall degradation by removing the acetyl group of xylan and catalyzing xyloglucan polymers, respectively, also causing berry ripening (Fig. 10).

## Conclusions

In this study, transcriptome analysis was used to investigate the molecular mechanism by which H_2_O_2_ treatment promotes the early ripening of Kyoho berries. Differentially expressed gene, TCseq, GO, KEGG, and WGCNA analyses were used to screen candidate genes and some key pathways. Some DEGs associated with berry ripening were identified, and included *HSP, GDSL, CAB1*, and *XTH* genes. The major genes were related to oxidative stress (*HSP* genes: including VIT_18s0089g01270, VIT_16s0022g00510, VIT_08s0058g00210, VIT_02s0154g00480, and VIT_01s0010g02290; functional annotation: heat shock protein), cell wall deacetylation (*GDSL* genes: including VIT_09s0002g00550 and VIT_05s0020g04840; functional annotation: GDSL esterase/lipase), cell wall degradation (*XTH* genes: including VIT_05s0062g00250 and VIT_02s0012g02220; functional annotation: xyloglucan endotransglucosylase/hydrolase), and photosynthesis (*CAB1* gene: VIT_10s0003g02890; functional annotation: chlorophyll a-b binding protein). In addition, the TCseq, KEGG, and WGCNA analyses revealed that photosynthesis was a key pathway during berry ripening. In summary, RNA-sequencing analysis indicated that H_2_O_2_ treatment promoted Kyoho berry early ripening by affecting the expression levels of these genes and pathways.

## Methods

### Plant material

Five-year-old Kyoho vines were obtained from Yanshi County, Luoyang, China (34.41°N, 112.46°E), and were cultivated from cuttings at the experimental farm of Henan University of Science and Technology. The berries were identified and stored in the laboratory. Field management was conducted according to local agricultural practices. The concentration of 30% H_2_O_2_ was 300 mmol/L. Distilled water was used for dilution, and the surfactant was Silwet-77 (Solarbio, Beijing, China) added at 300 μL/L. Twenty milliliters of the solution was sprayed evenly on the young berries of each cluster twice, and the treatment method followed that described in a previous study [10]. The first and second sprayings were applied at 25 and 35 days post anthesis (dpa), respectively, in 2017. The control received 20 mL of distilled water with 300 μL/L of Silwet-77. Each treatment was replicated three times. Thirty bunches were sampled from five vines, with the 30 bunches as one replication. The information on the sampling time points is provided in Table 2.

**Table 2:** Sampling time points of Kyoho bunches treated with or without hydrogen peroxide in 2017

Days post anthesis				
Treatment sampling time	35 (H1)	45 (H2)	55 (H3)	65 (H4)
Control sampling time	35 (C1)	45 (C2)	55 (C3)	65 (C4)

Note: The sprayings were conducted on June 13 (35 dpa) and June 23 (45 dpa), respectively

### Total RNA extraction, library construction, and RNA sequencing

Total RNA was isolated from Kyoho grape berries in the treatment and control groups using an RNAprep Pure Plant Kit (Polysaccharides & Polyphenolics rich, DP441; Tiangen, Beijing, China) according to the manufacturer’s instructions. The RNA was extracted from whole berries without the seeds. The berries, 0.5 g, were ground, and 0.1 g of tissue was used for each extraction. The experimental method was according to the manufacturer’s instructions. RNA integrity was evaluated with an RNA Nano 6000 Assay Kit using a Bioanalyzer 2100 system (Agilent Technologies, CA, USA). Three biological replicates were analyzed.

The sequencing libraries were constructed using a NEBNext® Ultra™ RNA Library Prep Kit for Illumina® (NEB, USA) according to the manufacturer’s instructions. The mRNA was separated and purified from total RNA using magnetic beads oligo (dT). A total of 3 μg of mRNA per sample was used as material for library construction. The purified mRNA was fragmented using fragmentation buffer. Then, the first-strand cDNA and the second strand were biosynthesized and purified with a QIAQuick PCR kit (Beijing Zhijie Fangyuan Technology Co. Ltd., China), and EB buffer was added for elution. After elution and purification, the double-stranded cDNA was repaired at the ends and the base ‘A’ and the sequencing joint were added. The target fragments were recovered with agarose gel electrophoresis. The PCR products were purified with an AMPure XP system (Beckman Coulter, Indianapolis, IN, USA), and the library quality was assessed with an Agilent 2100 Bioanalyzer. Three biological replicates were sequenced for each sample on an Illumina HiSeq™ 2500 platform at the Anoroad Genome Technologies Co., Ltd. (Beijing, China).

### Sequence data filtering, de novo assembly, and annotation

After trimming the adaptor sequences and removing poly-N reads and low-quality sequences with more than 50% bases and Q_phred_ ≤ 19 from the raw data of RNA sequencing, the clear reads were assembled by HISAT2 with default parameters. Bowtie (1.0.1 version) was used to build the index of the reference genome, and HISAT2 (2.1.0 version; Kim et al. [99]) was used to map the clean reads to the reference genome (ftp://ftp.ensemblgenomes.org/pub/plants/release-38/fasta/vitis_vinifera). To functionally annotate genes, all assembled transcripts were mapped to sequences available in public databases, including the non-redundant protein (NR) (http://www.ncbi.nlm.nih. gov/) and Swiss-Prot [100], Protein family (Pfam) [101], Gene Ontology (GO) [102], Clusters of Orthologous Groups (COG) [103], euKaryotic Orthologous Groups (KOG) [104] and Kyoto Encyclopedia of Genes and Genomes (KEGG) [105] databases.

### Cluster and differentially expressed gene analyses

Following alignments, raw counts of total genes were normalized to FPKM. According to the gene expression level, two was used as the log base. The value of mean expression was calculated at different berry developmental stages for each expressed gene. The TCseq clustering method was used to characterize the expression patterns of total genes [106].

The gene expression levels were quantified with FPKM values. HTseq (0.6.0 version) was used to count the number of clean reads mapped to each predicted gene. The DESeq2 [107] R package (1.6.3 version, TNLIST, Beijing, China) was used to identify differentially expressed genes based on the read count of each gene at different developmental stages. The resulting *P*-values were adjusted using the Benjamini and Hochberg’s approach. Genes with an adjusted *P*-value ≤0.05 and |log2 fold change| ≥ 1 revealed by DESeq2 were assigned as differentially expressed genes (DEGs).

### Weighted gene co-expression network analysis

Weighted gene co-expression network analysis (WGCNA) was also used to evaluate gene expression. The WGCNA R package [108] was used to appraise modules of correlated genes and to investigate intramodular hub genes [109]. The eigengene value of each module was evaluated to examine the association with each gene [110]. The hub genes of each model were verified by calculating the connectivity degree (number of neighbors) of each gene with Cytoscape v3.6.1 [111]. The eigengene value of each module was calculated to test the association with each tissue type. The hub genes were defined by kME > 1, which measures a gene’s connectivity in the specific module. To further identify those modules, GO and KEGG enrichment analyses were performed with the ClusterProfiler package [112].

### Quantitative real-time PCR

To confirm the DEG and hub gene results in the RNA-sequencing data, 14 DEGs and one hub gene were selected for further RT-qPCR measurement on a Bio-Rad real-time PCR system (CFX96 Touch, USA) using a TransStart Top Green qPCR SuperMix kit (Transgen, Beijing China). Total RNA was isolated from ‘Kyoho’ grape berries in the treatment and control groups using an RNAprep Pure Plant Kit (Polysaccharides & Polyphenolics rich, DP441; Tiangen, Beijing, China) according to the manufacturer’s instructions. The RNA was extracted from whole berries without the seeds. The berries, 0.5 g, were ground, and 0.1 g of tissue was used for each extraction. The experimental method was according to the manufacturer’s instructions. RNA integrity was evaluated with an RNA Nano 6000 Assay Kit using a Bioanalyzer 2100 system (Agilent Technologies, CA, USA). A HiScript II 1st Strand cDNA Synthesis Kit (R211–01; Vazyme, Nanjing, China) was used for reverse transcription. The reverse transcription was performed at 25 °C for 5 min, 52 °C for 15 min, and 85 °C for 5 min. The primers of the 15 genes were constructed using Primer Premier 5.0 software, and the sequences are provided in Supplemental Table S4. The suitable reverse-transcribed cDNA was used as the template for RT-qPCR measurement. The reaction was performed in 10-μL assays that contained 1 μL of cDNA, 5 μL of 2× Trans Start® Top Green qPCR SuperMix, 0.3 μL of each primer, and 3.4 μL of added double-distilled water. Reactions were performed in 40 cycles of 94 °C for 30 s, 94 °C for 5 s, and 60 °C for 30 s. Three biological and technical replicates were analyzed for each sample. The ubiquitin gene served as the reference gene. All analyses included three independent biological replicates. The relative expression levels of the target genes were calculated using the 2^−ΔΔCT^ approach [113].

### Statistical analyses

The RT-qPCR results of the 15 genes at single time points were compared by one-way ANOVA followed by a Tukey’s HSD post-hoc test with SPSS software (21.0 version, IBM Corp., NY, USA). Data are presented as means ± SD. The asterisk (*) stands for the levels of significant difference (*p value ≤0.05, **p value ≤0.01).

## Supplementary Information

**Additional file 1: Supplemental Table S1.** Data overview of the transcriptome sequencing.

**Additional file 2: Supplemental Table S2.** The gene number and gene annotation information of DEGs from the comparison of H4-K4 stage.

**Additional file 3: Supplemental Table S3.** The node genes and hub gene annotation from the WGCNA analysis shown in Fig. 7.

**Additional file 4: Supplemental Table S4.** Primers used for quantitative PCR analysis in this study.

**Additional file 5: Supplemental Table S5.** The numerical outputs of qRT-PCR with Tukey's HSD post hoc test. The asterisk (*) and bold values stands for the levels of significant difference (*p value ≤ 0.05, **p value ≤ 0.01).

**Additional file 6: Supplemental Figure S1.** Grouping optimization of gene expression patterns for TC-seq analysis based on Calinski criterion value.

**Additional file 7: Supplemental Figure S2.** Cytoscape representation of co-expressed genes with edge weight ≥ 0.10. The important hub gene was noted with yellow. **a** The hub gene of H1 stage; **b** The hub gene of K1 stage; **c** The hub gene of K3 stage; **d** The hub gene of K4 stage, respectively.

**Additional file 8: Supplemental Figure S3.** Correlation between the gene expression ratios obtained from RNA-seq data and qRT-PCR. The x-axis indicates the log2 transformed FPKM of genes at different development stages. The y-axis represents the log2 transformed expression of genes at different development stages.

**Additional file 9: Supplemental Figure S4.** Scattergram of both GO enrichment analysis and KEGG pathways analysis. The X-axis indicates the berry development stages; the Y-axis indicates the GO terms or KEGG pathway. a GO enrichment analysis of WGCNA; b KEGG pathway analysis of WGCNA. Coloring indicates q-value with higher in red and lower in blue. And the lower q-value indicates the more significantly enriched. Point size indicates genes number.

## Data Availability

The RNA-Seq data supporting the results of this article have been uploaded to the Sequence Read Archive of NCBI (National Center for Biotechnology Information). It could be accessed via the NCBI SRA database with accession numbers of PRJNA541089 from 5th May 2019 onwards. Until then, these sequences are available from the corresponding author upon reasonable request.

## Abbreviations

CAB: Chlorophyll a-b binding protein
COG: Clusters of Orthologous Groups
DEGs: Differentially expressed genes
dpa: Days post anthesis
FPKM: Fragments Per Kilobase per Million
GLIP: GDSL esterase/lipase
GSTF12: Glutathione S-transferase F12
GO: Gene Ontology
H_2_O_2_: Hydrogen peroxide
HSP: Heat shock protein
KEGG: Kyoto Encyclopedia of Genes and Genomes
KOG: Karyotic Orthologous Groups
LAC14: Laccase-14
O_2_$$ \overline{\cdotp} $$: Superoxide anion
OMT1: Caffeic acid 3-O-Methyltransferase
PAP15: Purple acid phosphatase
PCA: Principal component analysis
PCD: Programmed cell death
PSII: Photosystem II
PSA: Photosystem I reaction center subunit II
qRT-PCR: quantitative real-time reverse transcription PCR
RNA-seq: RNA-sequencing
ROS: Reactive oxygen species
TCseq: Time Course sequencing
WGCNA: Weighted gene co-expression network analysis
XTH30: Xyloglucan endotransglucosylase/hydrolase protein 30
