# Supplementary material for: Transcriptome analysis reveals mechanism of early ripening in Kyoho grape with hydrogen peroxide treatment
Source: BMC Genomics. 2020 Nov 11;21:784. doi: 10.1186/s12864-020-07180-y (PMC7657363; doi:10.1186/s12864-020-07180-y)
Supplement: Supplementary file 1 — Additional file 1: Supplemental Table S1. Data overview of the transcriptome sequencing. [file 12864_2020_7180_MOESM1_ESM.docx]

**Supplemental Table S1** Overview of the ‘Kyoho’ transcriptome sequencing.

| Sample | Raw Reads Number | Clean Reads Number | Low-quality Reads Number | Mapped Reads | Clean Reads Rate (%) | Mapping Rate (%) | Clean Q30 Bases Rate (%) |
| --- | --- | --- | --- | --- | --- | --- | --- |
| K11 | 49,138,104 | 47,666,142 | 699,516 | 43,452,805 | 97.00 | 91.16 | 94.22 |
| K12 | 48,485,058 | 46,916,240 | 663,158 | 43,339,437 | 96.76 | 92.38 | 93.75 |
| K13 | 49,139,946 | 47,812,104 | 587,404 | 43,913,218 | 97.30 | 91.85 | 94.78 |
| K22 | 46,459,288 | 44,923,334 | 498,704 | 41,160,758 | 96.69 | 91.62 | 95.01 |
| K23 | 41,257,008 | 39,598,742 | 322,970 | 35,728,491 | 95.98 | 90.23 | 94.29 |
| K31 | 48,900,572 | 47,321,168 | 599,608 | 42,936,921 | 96.77 | 90.74 | 94.56 |
| K32 | 47,079,342 | 45,284,260 | 395,258 | 41,317,932 | 96.19 | 91.24 | 94.96 |
| K33 | 46,539,148 | 45,143,926 | 333,072 | 40,960,215 | 97.00 | 90.73 | 95.19 |
| K41 | 47,967,094 | 45,716,536 | 604,624 | 41,200,912 | 95.31 | 90.12 | 93.90 |
| K42 | 49,356,126 | 47,308,414 | 690,302 | 42,841,875 | 95.85 | 90.56 | 94.09 |
| K43 | 48,221,786 | 47,051,192 | 386,558 | 42,681,062 | 97.57 | 90.71 | 94.74 |
| H11 | 50,027,308 | 47,953,772 | 563,000 | 43184892 | 95.86 | 90.06 | 94.76 |
| H12 | 48,115,484 | 46,071,628 | 521,228 | 42545699 | 95.75 | 92.35 | 94.80 |
| H13 | 48,978,472 | 47,636,026 | 401,356 | 44001020 | 97.26 | 92.37 | 95.14 |
| H21 | 48,009,402 | 46,280,760 | 475,558 | 42144377 | 96.40 | 91.06 | 94.53 |
| H22 | 46,224,364 | 44,993,820 | 411,926 | 41148839 | 97.34 | 91.45 | 95.04 |
| H23 | 46,344,252 | 45,045,540 | 527,078 | 41004561 | 97.20 | 91.03 | 94.55 |
| H31 | 48,094,916 | 45,483,400 | 765,618 | 40458831 | 94.57 | 88.95 | 92.20 |
| H32 | 46,166,298 | 44,951,074 | 550,908 | 40476683 | 97.37 | 90.05 | 94.26 |
| H33 | 45,975,428 | 44,291,056 | 418,602 | 40187551 | 96.34 | 90.74 | 94.98 |
| H41 | 47,908,074 | 46,522,110 | 428,424 | 41615330 | 97.11 | 89.45 | 94.83 |
| H42 | 48,070,388 | 46,761,774 | 470,782 | 42456885 | 97.28 | 90.79 | 94.82 |
| H43 | 46,364,272 | 44,917,446 | 507,928 | 40216227 | 96.88 | 89.53 | 94.34 |
