# Supplementary material for: Transcriptome analysis reveals mechanism of early ripening in Kyoho grape with hydrogen peroxide treatment
Source: BMC Genomics. 2020 Nov 11;21:784. doi: 10.1186/s12864-020-07180-y (PMC7657363; doi:10.1186/s12864-020-07180-y)
Supplement: Supplementary file 4 — Additional file 4: Supplemental Table S4. Primers used for quantitative PCR analysis in this study. [file 12864_2020_7180_MOESM4_ESM.docx]

**Supplemental Table S4** Primers used for quantitative PCR analysis in this study.

| Gene name | | Gene ID | |  | | Sequence (5’- 3’) | |
| --- | --- | --- | --- | --- | --- | --- | --- |
| *LAC14* | | VIT_18s0164g00100 | | Forward | | CATAGCCTGACTGTTGTTGG | |
|  |  |  |  | Reverse | | GTAGTGACTAGGAGCCTGAT | |
| *GLIP* | | VIT_09s0002g00550 | | Forward | | TGTTCGTCTTTGGCGATTCC | |
|  |  |  |  | Reverse | | TTCCAGGATGAGTGTCAGCAAG | |
| *GSTF12* | | VIT_04s0079g00690 | | Forward | | CATGCAGCTAGTGATCCTAC | |
|  |  |  |  | Reverse | | CAGATCAGCGAGAGTGAATG | |
| *XTH15* | | VIT_05s0062g00250 | | Forward | | CTATGGAATGCGGATGACTG | |
|  |  |  |  | Reverse | | GAATGAGTTTGAGGAGGTGGAG | |
| *XTH30* | | VIT_02s0012g02220 | | Forward | | GCAATGACTATTCCGCCATC | |
|  |  |  |  | Reverse | | GATGATGACACACTCCGGTAG | |
| *HSP23* | | VIT_16s0022g00510 | | Forward | | AAGGTAAGCGTGGAGCAGAAC | |
|  |  |  |  | Reverse | | CACCTTTGTTCATCTCAGCC | |
| *HSP21* | | VIT_01s0010g02290 | | Forward | | GAGTGGGACTGAAGGAAATGG | |
|  |  |  |  | Reverse | | ATCTTGGGGTTGTCACTGGC | |
| *VIT_208s0058g00210* | | VIT_08s0058g00210 | | Forward | | CTTCGCAGATTCAGACTTCC | |
|  |  |  |  | Reverse | | GCTTCTTCACCTCCTCTTTCG | |
| *ATHSP22* | | VIT_18s0089g01270 | | Forward | | TCTTTGCCCAACTTGTCGTC | |
|  |  |  |  | Reverse | | GTTCCTGTTTGGAGGTTTCAC | |
| *GDSL* | | VIT_05s0020g04840 | | Forward | | TGGAGTGGTCTGTGATCCTT | |
|  |  |  |  | Reverse | | CTCATTCACCCTCACTGCTC | |
| *CAB1* | | VIT_10s0003g02890 | | Forward | | CATGTTCGGGTTCTTCGTTC | |
|  |  |  |  | Reverse | | TCCAGGCGTTGTTGTTGAC | |
| *OMT1* | VIT_15s0048g02450 | | Forward | | GATAGCATCCCACTTAGCCAC | |  |
|  |  |  | Reverse | | TGGAGTGCCCATCATCAGT | |  |
| *VIT_217s0000g00430* | VIT_17s0000g00430 | | Forward | | TGAAGGATGGAGAAGAGGAG | |  |
|  |  |  | Reverse | | CTTCTTACCCTCTCTGCAAG | |  |
| *HSP23* | VIT_02s0154g00480 | | Forward | | CACACTCATCATCAAAGGCG | |  |
|  |  |  | Reverse | | CACCACCTTCAGTACACCAT | |  |
| *PAP* | VIT_01s0011g06290 | | Forward | | TGACTATGACTGGAAGGGCGT | |  |
|  |  |  | Reverse | | TTTGCCTGAAGGATTGGGAG | |  |
