# Supplementary material for: Transcriptome analysis reveals mechanism of early ripening in Kyoho grape with hydrogen peroxide treatment
Source: BMC Genomics. 2020 Nov 11;21:784. doi: 10.1186/s12864-020-07180-y (PMC7657363; doi:10.1186/s12864-020-07180-y)
Supplement: Supplementary file 5 — Additional file 5: Supplemental Table S5. The numerical outputs of qRT-PCR with Tukey's HSD post hoc test. The asterisk (*) and bold values stands for the levels of significant difference (*p value ≤ 0.05, **p value ≤ 0.01). [file 12864_2020_7180_MOESM5_ESM.docx]

**Supplemental Table S5** The numerical outputs of qRT-PCR with Tukey's HSD post hoc test. The asterisk (*) and bold values stands for the levels of significant difference (*p value ≤ 0.05, **p value ≤ 0.01).

| Gene ID | Days post anthesis (d) | Tukey's HSD hoc test |
| --- | --- | --- |
| XTH15 | 35 | 0.972939 |
|  | 45 | 0.373697 |
|  | 55 | **0.003311**** |
|  | 65 | **0.003297**** |
| XTH30 | 35 | 0.428757 |
|  | 45 | 0.071596 |
|  | 55 | **0.035335*** |
|  | 65 | **8.92E-05**** |
| CAB1 | 35 | **0.006553**** |
|  | 45 | 0.647557 |
|  | 55 | **0.024356*** |
|  | 65 | **0.000358**** |
| HSP21 | 35 | **2.04E-06**** |
|  | 45 | 0.77894 |
|  | 55 | **1.81E-07**** |
|  | 65 | **0.000334**** |
| ATHSP22 | 35 | **0.002675**** |
|  | 45 | 0.158386 |
|  | 55 | 0.328263 |
|  | 65 | 0.060226 |
| HSP23 | 35 | **0.049607*** |
|  | 45 | 0.449442 |
|  | 55 | **0.005475**** |
|  | 65 | **0.049607*** |
| HSP23 | 35 | **8.88E-05**** |
|  | 45 | 0.471194 |
|  | 55 | **0.005586**** |
|  | 65 | **0.000221**** |
| PAP | 35 | **0.000396**** |
|  | 45 | **0.010191*** |
|  | 55 | 0.727545 |
|  | 65 | **2.4E-05**** |
| OMT1 | 35 | **0.001186**** |
|  | 45 | **0.030266*** |
|  | 55 | 0.348654 |
|  | 65 | 0.957561 |

| Gene ID | Days post anthesis (d) | Duncan’s post hoc test |
| --- | --- | --- |
| GLP | 35 | **0.000102**** |
|  | 45 | **0.00955**** |
|  | 55 | **0.04902*** |
|  | 65 | **0.004077**** |
| GSTF12 | 35 | **3.14E-05**** |
|  | 45 | **0.002752**** |
|  | 55 | **0.002823**** |
|  | 65 | **0.002646**** |
| LAC14 | 35 | **0.000106**** |
|  | 45 | 0.062602 |
|  | 55 | **0.009111**** |
|  | 65 | **0.012447*** |
| VIT_05s0020g04840 | 35 | **4.78E-05**** |
|  | 45 | **0.008273**** |
|  | 55 | 0.566511 |
|  | 65 | **4.86E-06**** |
| VIT_08s0058g00210 | 35 | **9.37E-14**** |
|  | 45 | **0.000137**** |
|  | 55 | **0.000532**** |
|  | 65 | **9.39E-14**** |
| VIT_17s0000g00430 | 35 | 0.639872 |
|  | 45 | **0.042914*** |
|  | 55 | **0.000915**** |
|  | 65 | 0.202776 |
