# Supplementary figures and images for: Transcriptome analysis reveals mechanism of early ripening in Kyoho grape with hydrogen peroxide treatment
Source: BMC Genomics. 2020 Nov 11;21:784. doi: 10.1186/s12864-020-07180-y (PMC7657363; doi:10.1186/s12864-020-07180-y)

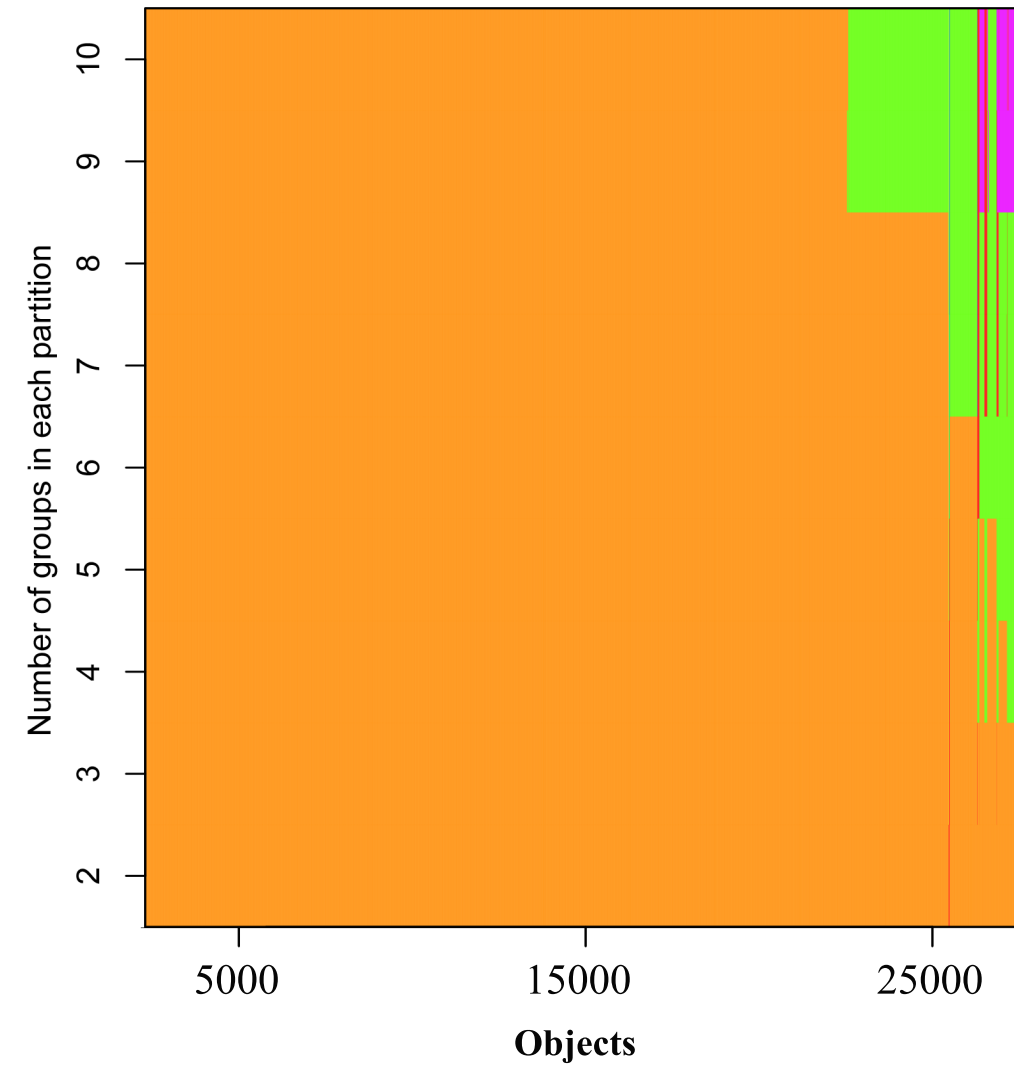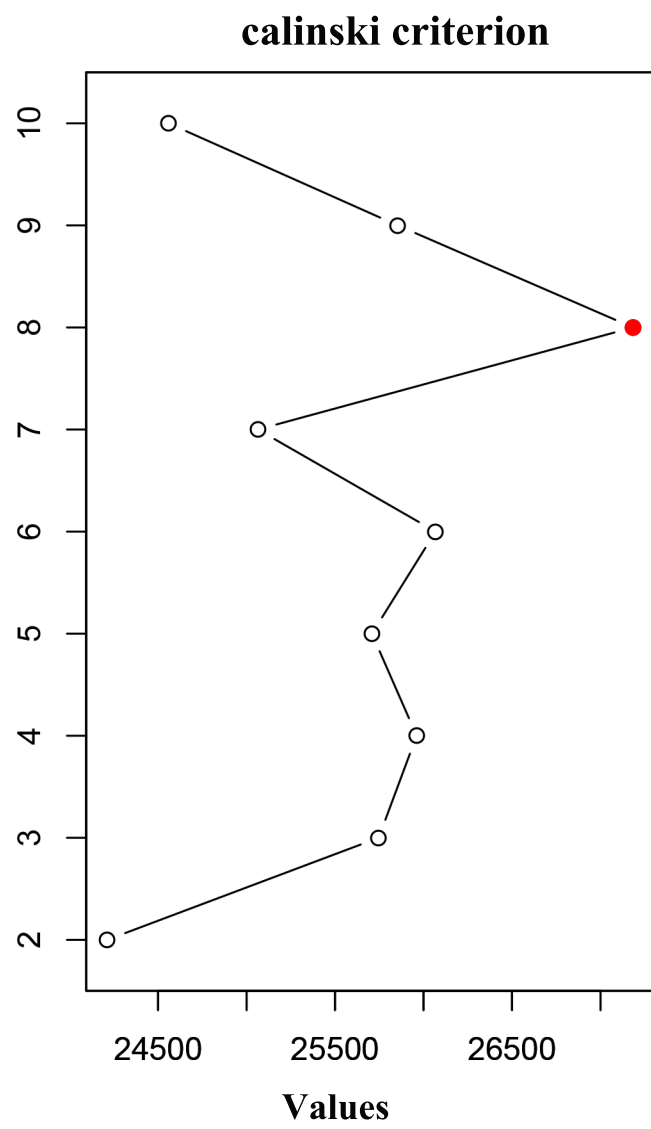

Supplement: Supplementary file 6 — Additional file 6: Supplemental Figure S1. Grouping optimization of gene expression patterns for TC-seq analysis based on Calinski criterion value. [file 12864_2020_7180_MOESM6_ESM.pdf]

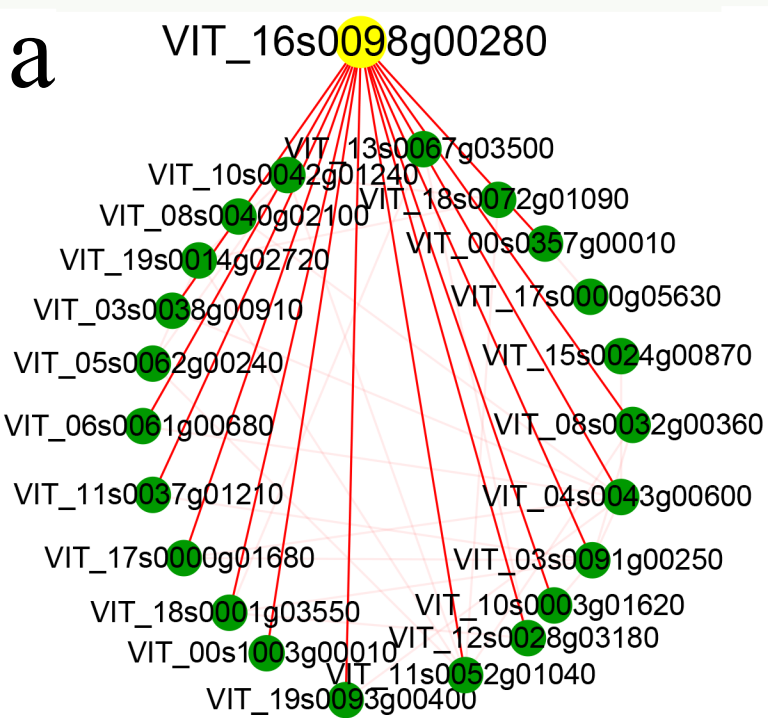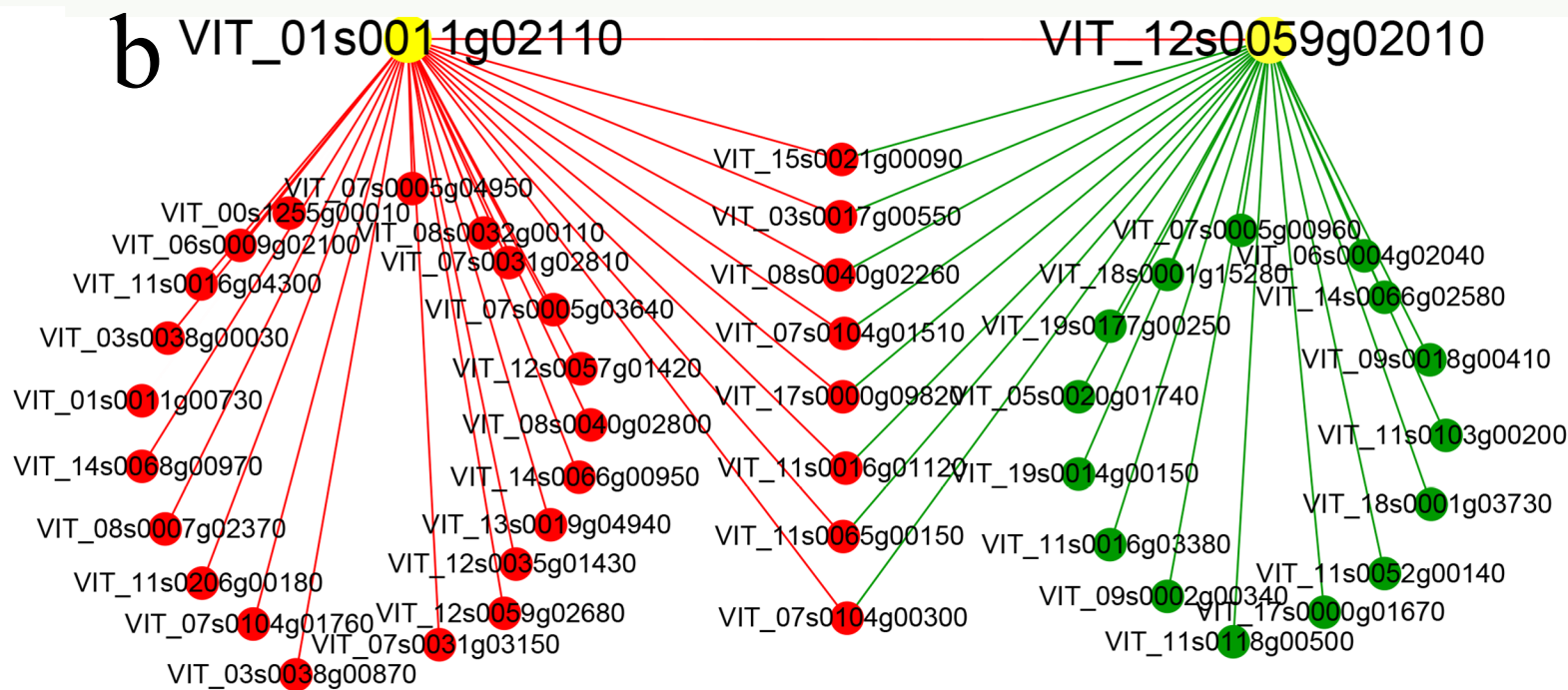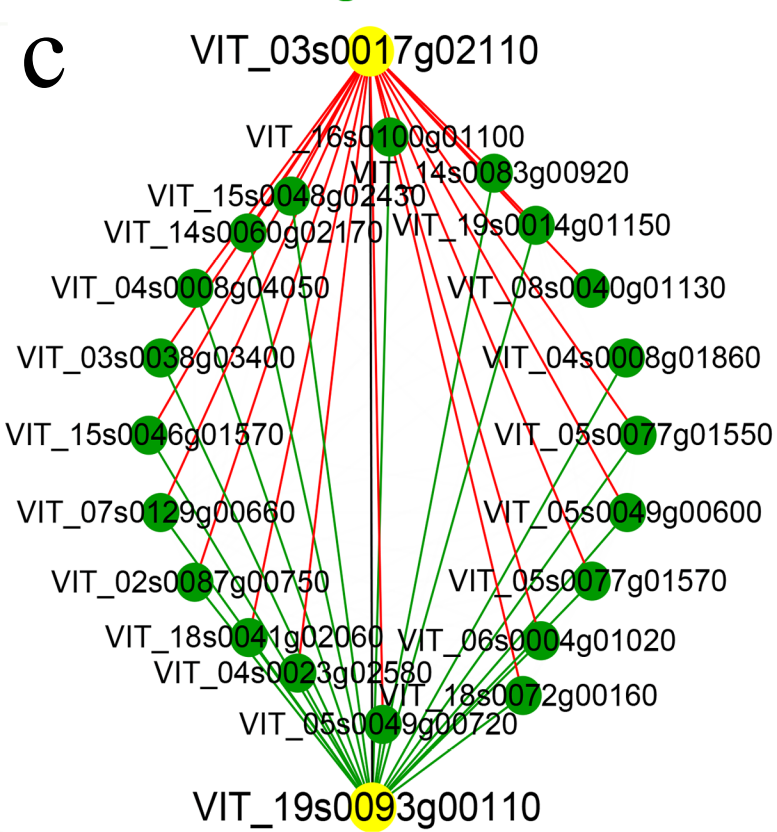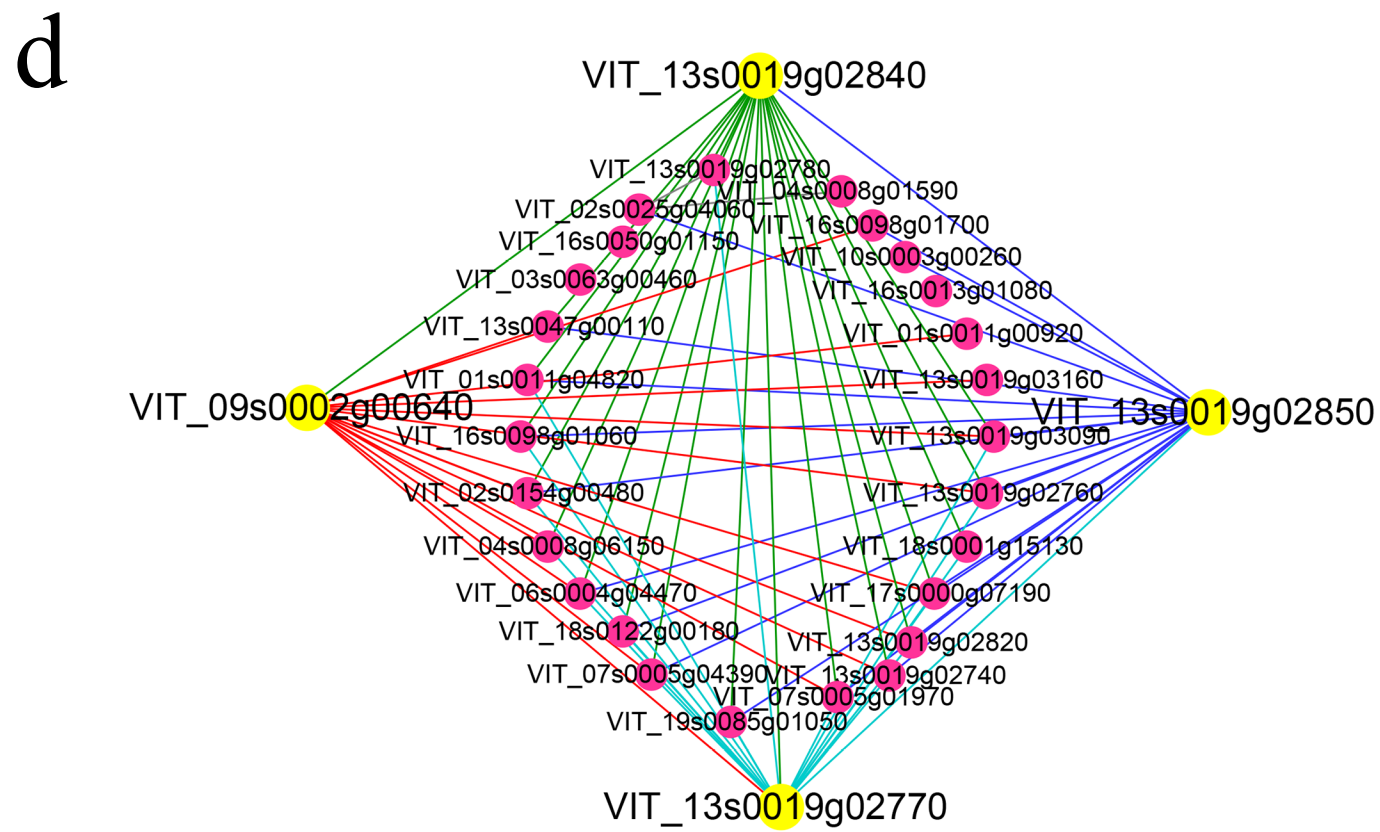

Supplement: Supplementary file 7 — Additional file 7: Supplemental Figure S2. Cytoscape representation of co-expressed genes with edge weight ≥ 0.10. The important hub gene was noted with yellow. a The hub gene of H1 stage; b The hub gene of K1 stage; c The hub gene of K3 stage; d The hub gene of K4 stage, respectively. [file 12864_2020_7180_MOESM7_ESM.pdf]

a

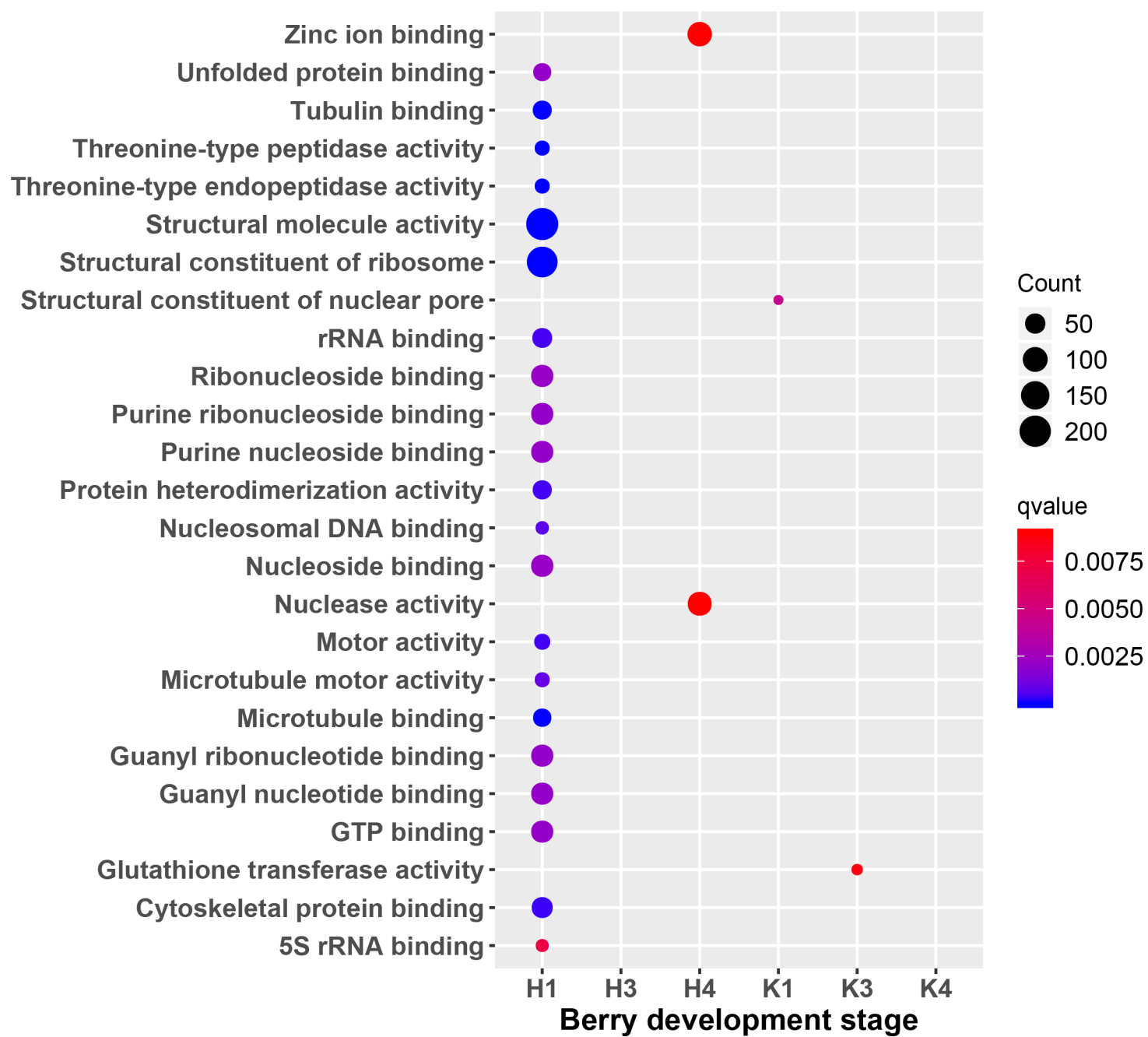

b

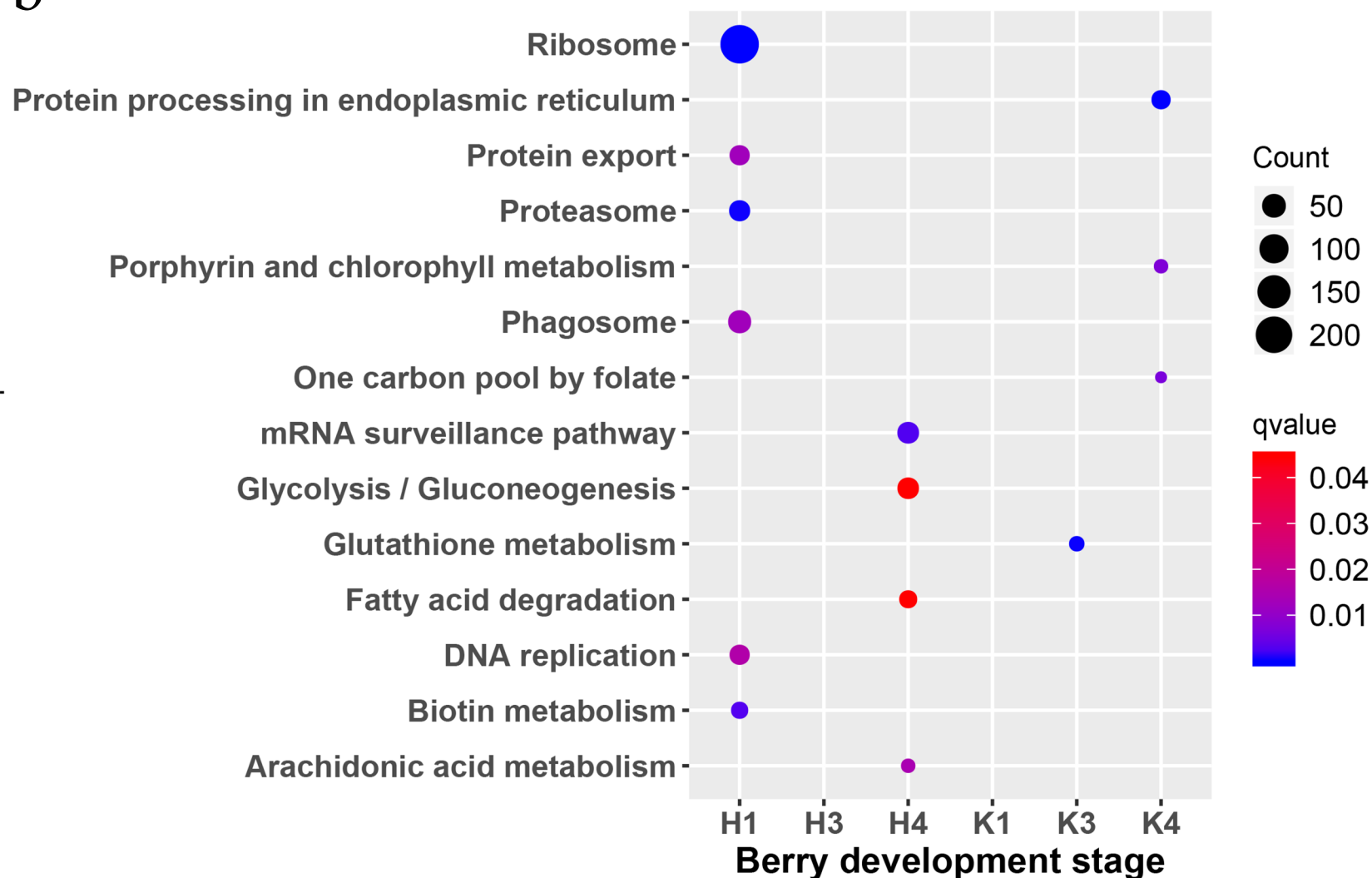

Supplement: Supplementary file 9 — Additional file 9: Supplemental Figure S4. Scattergram of both GO enrichment analysis and KEGG pathways analysis. The X-axis indicates the berry development stages; the Y-axis indicates the GO terms or KEGG pathway. a GO enrichment analysis of WGCNA; b KEGG pathway analysis of WGCNA. Coloring indicates q-value with higher in red and lower in blue. And the lower q-value indicates the more significantly enriched. Point size indicates genes number. [file 12864_2020_7180_MOESM9_ESM.pdf]
