# Supplementary material for: Transcriptome analysis reveals mechanism of early ripening in Kyoho grape with hydrogen peroxide treatment
Source: BMC Genomics. 2020 Nov 11;21:784. doi: 10.1186/s12864-020-07180-y (PMC7657363; doi:10.1186/s12864-020-07180-y)
Supplement: Supplementary file 8 — Additional file 8: Supplemental Figure S3. Correlation between the gene expression ratios obtained from RNA-seq data and qRT-PCR. The x-axis indicates the log2 transformed FPKM of genes at different development stages. The y-axis represents the log2 transformed expression of genes at different development stages. [file 12864_2020_7180_MOESM8_ESM.pdf]

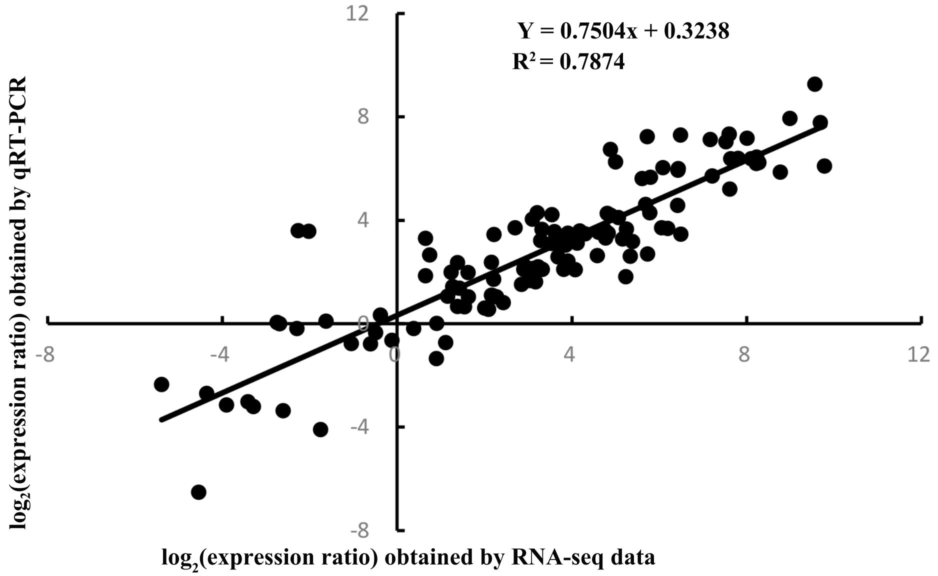

Y = 0.7504x + 0.3238
R² = 0.7874
log2(expression ratio) obtained by qRT-PCR
log2(expression ratio) obtained by RNA-seq data
12
8
4
0
-4
-8
-8
-4
0
4
8
12
